# Contribution of Small RNA Pathway to Interactions of Rice with Pathogens and Insect Pests

**DOI:** 10.1186/s12284-021-00458-z

**Published:** 2021-02-06

**Authors:** Qin Feng, Yan Li, Zhi-Xue Zhao, Wen-Ming Wang

**Affiliations:** Rice Research Institute and Research Center for Crop Disease and Insect Pests, Sichuan Agricultural University at Wenjiang, 211 Huimin Road, Wenjiang District, Chengdu, 611130 China

**Keywords:** MicroRNA, Small interfering RNA, ARGONAUTE, Dicer-like, RNA dependent RNA polymerase, Rice immunity, Hormone signal

## Abstract

Small RNAs (sRNAs) are mainly classified into microRNAs (miRNAs) and small interfering RNAs (siRNAs) according to their origin. miRNAs originate from single-stranded RNA precursors, whereas siRNAs originate from double-stranded RNA precursors that are synthesized by RNA-dependent RNA polymerases. Both of single-stranded and double-stranded RNA precursors are processed into sRNAs by Dicer-like proteins. Then, the sRNAs are loaded into ARGONAUTE proteins, forming RNA-induced silencing complexes (RISCs). The RISCs repress the expression of target genes with sequences complementary to the sRNAs through the cleavage of transcripts, the inhibition of translation or DNA methylation. Here, we summarize the recent progress of sRNA pathway in the interactions of rice with various parasitic organisms, including fungi, viruses, bacteria, as well as insects. Besides, we also discuss the hormone signal in sRNA pathway, and the emerging roles of circular RNAs and long non-coding RNAs in rice immunity. Obviously, small RNA pathway may act as a part of rice innate immunity to coordinate with growth and development.

## Background

Plants employ a sophisticated immune system to protect themselves from biotic stress. The cell surface-located receptors surveil and recognize pattern molecules from pathogens to initiate immune responses, termed pattern-triggered immunity (PTI). Typical responses in PTI include the burst of reactive oxygen species (ROS), the induction of pathogenesis-related genes (*PR* genes), and the deposition of callose. Pathogens usually overcome PTI with effectors to facilitate their invasion and proliferation. In turn, plant resistance (R) proteins specifically recognize cognate effectors to activate immune responses, called effector-triggered immunity (ETI). Immune responses in ETI are much stronger than those in PTI and are often accompanied by the hypersensitive response (HR) at the invasion site (Boller and Felix 2009; Boller and He 2009; Jones and Dangl 2006).

Plant immune responses are tightly controlled by various immunity-associated regulators, such as transcription factors and small RNAs (sRNAs) (Chandran et al. 2018; Wang et al. 2018a). Plant endogenous sRNAs include miRNAs and siRNAs, both of them are processed by Dicer-like proteins (DCLs) and incorporated into ARGONAUTE proteins (AGOs) to form the RNA-induced gene silencing complexes (RISCs) (Baulcombe 2004). The RISCs specifically bind to DNA or RNA sequences of sRNAs target genes and repress their expression on transcriptional, post-transcriptional or translational levels through DNA methylation, mRNA cleavage or translational repression, respectively (Brodersen et al. 2008; Llave et al. 2002; Song et al. 2019; Wu et al. 2010). Obviously, DCLs and AGOs are two types of core components shared by miRNA and siRNA signaling pathway. DCLs are a kind of endoribonucleases that specifically slice double-stranded RNAs (dsRNAs) into sRNA duplexes (Carmell and Hannon 2004). AGOs recognize and incorporate the mature sRNAs to execute the sRNA-guided repression of target genes (Fabian et al. 2010; Hutvagner and Simard 2008; Wu et al. 2010). Besides, the biosynthesis of siRNAs requires the activity of RNA-dependent RNA polymerases (RDRs). The RDRs exploit the single-stranded RNA (ssRNA) transcripts as the templates to synthesize the long dsRNAs that act as the precursors of siRNAs (Wassenegger and Krczal 2006). The exogenous virus-derived siRNAs (vsiRNAs) are a type of well-studied triggers of antiviral defense. The biosynthesis and function of vsiRNAs are also dependent on host DCLs, RDRs and AGOs (Brosseau and Moffett 2015; Du et al. 2007; Qu et al. 2008). Plants employ the vsiRNAs to initiate gene silencing of virus transcripts, thus disturbing virus replication and proliferation, which is regarded as the plant self-defensive mechanism against viruses (Llave 2010; Yang and Li 2018). However, in some cases, vsiRNAs are indicated to direct RNA silencing of host genes to facilitate infection (Smith et al. 2011; Xia et al. 2018; Yang et al. 2020a).

**Fig. 1:**
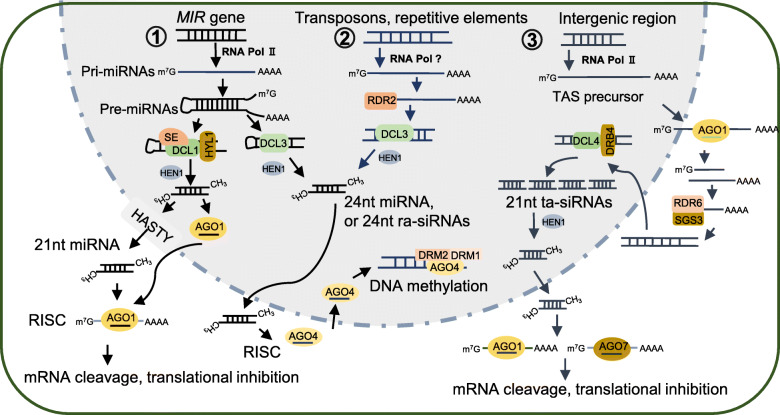
A simple model describes the biosynthesis and assembly of small RNAs. ① *MIR* genes are transcribed by RNA Polymerase II (RNA Pol II) into primary miRNAs (pri-miRNAs). Pri-miRNAs form an imperfect fold-back structure and then are processed into a stem-loop-structured precursor miRNAs (pre-miRNAs). The pre-miRNAs are further processed into 21- or 24-nt miRNA duplexes by the complex that mainly includes DCL1 or DCL3, HYPONASTICLEAVES1(HYL1) and SERRATE (SE). The overhang ends of miRNA duplex are methylated by HUA ENHANCER 1 (HEN1) to increase stability and then one strand of the miRNA duplex is loaded into AGO proteins to form RNA-induced gene silencing complex (RISC) in the cytoplasm or the nucleus. The 21-nt miRNAs exploit two pathways to incorporate into AGO1 protein. One, the miRNA duplex is exported to the cytoplasm via HASTY channel, then one strand of the duplex is loaded into AGO1 to form RISC. Two, some 21-nt miRNAs could be directly loaded into AGO1 to form RISC in the nucleus and the RISC is exported to the cytoplasm. Then, the RISCs repress gene expression via mRNA cleavage, or translational inhibition. Besides, 24-nt miRNAs are loaded into AGO4 in the cytoplasm and reenter into the nucleus to mediate DNA methylation by recruiting DOMAINS REARRANGED METHYLTRANSFERASE (DRM). ② The transposons or repetitive elements are transcribed by an unknown RNA Polymerase (RNA Pol?), then these transcripts are used as templates by RDR2 to synthesize double stranded RNAs (dsRNAs). Later, the dsRNAs are processed into 24-nt repeat-associated siRNAs (ra-siRNAs) by DCL3 and are methylated by HEN1 at 3′-terminal. Then one strand of ra-siRNAs is loaded into AGO4 to form RISC to mediate DNA methylation of target gene by recruiting the methyltransferases DRM1 and DRM2. ③ The specific intergenic regions are transcribed by RNA Pol II to generate the TAS precursors. The TAS precursors are targeted by some specific miRNA-AGO1 complex (such as miR390-AGO1 or miR173-AGO1) and are cleaved into fragments, then the 5′ or 3′ fragments of TAS precursors are exploited by RDR6 and SGS3 as the templates to synthesize the dsRNAs. The dsRNAs are imported into the nucleus and processed by DCL4 and DRB4 into 21-nt phased, trans-acting siRNAs (ta-siRNAs). After the methylation of ta-siRNAs at 3′-terminal, ta-siRNAs are exported into the cytoplasm and one strand of the ta-siRNAs is loaded into AGO1 or AGO7 to form RISC to suppress gene expression

The biosynthesis of miRNAs is different from that of siRNAs (Fig. 1). miRNAs originate from the primary miRNA transcripts (pri-miRNAs) that are transcribed by RNA polymerase II (Pol II). Later, the single-stranded pri-miRNAs are processed into the precursor miRNAs (pre-miRNAs) that possess a stem-loop structure (He and Hannon 2004; Henderson et al. 2006). In contrast, siRNAs originate from multiple different transcripts that are transcribed by Pol II or Pol IV, such as TAS transcripts, cis-antisense overlapping coding transcripts, and transcripts derived from the retroelements or repetitive DNA (Hamilton et al. 2002; Katiyaragarwal and Jin 2010; Onodera et al. 2005; Vazquez 2006; Yoshikawa et al. 2005). All of these transcripts are exploited by RDRs to synthesize the double-stranded siRNA precursors (Wassenegger and Krczal 2006). The pre-miRNAs and the double-stranded siRNA precursors are sliced into sRNA duplexes by DCLs, then one strand of the sRNA duplex is incorporated into AGOs to form RISCs (Carmell and Hannon 2004; Song et al. 2019; Voinnet 2009). Besides, the processing of vsiRNAs is similar to that of the endogenous siRNAs. The production of vsiRNAs is divided into primary vsiRNAs and secondary vsiRNAs (Llave 2010). The primary vsiRNAs are derived from dsRNAs that are generated by viral RDRs during genome replication and transcription (RNA viruses) or via converging bidirectional transcription (DNA viruses), and the dsRNAs are perceived by the host DCLs to cut into sRNAs duplexes. Whereas, the secondary vsiRNAs are produced from dsRNAs that are synthesized by the host RDRs from the aberrant viral RNA cleavage products. Both the primary vsiRNAs and the secondary vsiRNAs are loaded into the host AGOs to form the RISCs (Aliyari et al. 2008; Llave 2010; Qu et al. 2008).

**Fig. 2:**
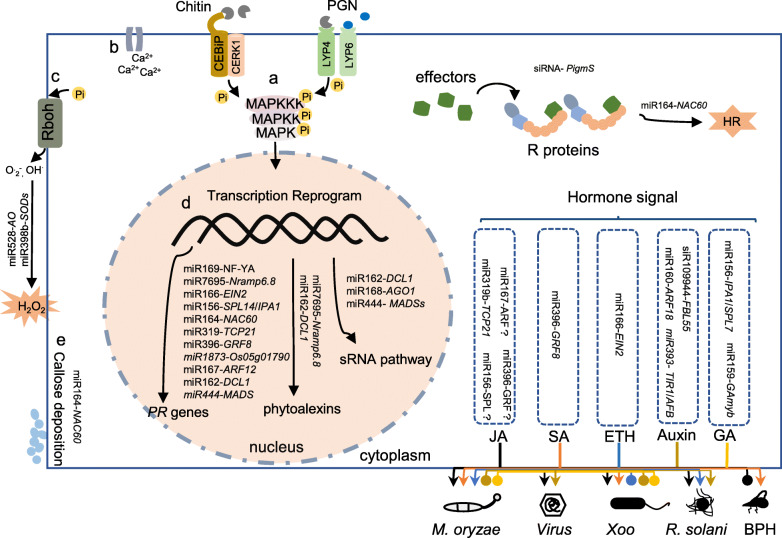
Small RNA regulatory modules in PTI, ETI, and hormone signal pathway. PAMPs derived from fungi or bacteria, such as chitin or peptidoglycan (PGN), are recognized by the pattern-recognition receptors (i.e. CEBiP and CERK1, or LYP4, and LYP6) to activate PTI responses including: **a**) activation of MAPK cascades, **b**) calcium influx, **c**) ROS burst, **d**) the induction of *PR* genes and phytoalexins genes, and e) callose deposition. The miR528-*AO* and miR398b-*SODs* modules contribute to the production of superoxide radicals (O·2^**−**^) and hydroxyl radical (OH^·^), and H_2_O_2_ accumulation. The modules regulating *PR* genes expression include miR169-*NF-YA4/10/11*, miR7695-*Nramp6.8*, miR166-*EIN2*, miR156-*SPL14*/*IPA1*, miR164-*NAC60*, miR319-*TCP21*, miR396-*GRF8*, miR1873-*Os05g01790*, miR167-*ARF12*, miR162-*DCL1*, and miR444-*MADSs*. The miR7695-*Nramp6.8* and miR162-*DCL1* module also participate in phytoalexins biosynthesis. The miR164-*NAC60* module may be involved in callose deposition. Besides, the recognition between effectors and R proteins activates ETI that often leads to the hypersensitive response (HR). The siRNAs derived from the miniature transposons MITE1 and MITE2 regulate *PigmS* expression thus control the activity of PigmR; and the miR164-*NAC60* module may contribute to the occurrence of HR. Hormone signal pathways differently regulate rice resistance to *Magnaporthe oryzae* (*M*. *oryzae*), viruses, *Xanthomonas oryzae* pv*. oryzae* (*Xoo*), *Rhizoctonia solani* (*R*. *solani*) and the planthopper (BPH). Jasmonic acid (JA) signal pathway positively regulates rice immunity against *M*. *oryzae*, viruses, *Xoo*, and *R. solani* but enhances susceptibility to BPH. Salicylic acid (SA) signal pathway positively regulates rice immunity against *M. oryzae*, *Xoo* and BPH. Ethylene (ET) signal pathway positively regulates rice immunity against *M*. *oryzae*, *R*. *solani* but enhances susceptibility to *Xoo*. Auxin signal pathway positively regulates rice immunity against virus and *R. solani* but increases susceptibility to *M. oryzae* and *Xoo*. Gibberellic acid (GA) signal pathway negatively regulates rice immunity against *M. oryzae* and *Xoo*. Four modules may block JA signal pathway to promote the infection of *M. oryzae*, or *Xoo*, including miR167-*ARFs*, miR396-*GRFs*, miR319-*TCP21*, and miR156-*SPLs*. The miR396-*GRF8* module may impair SA signal pathway to promote the infestation of BPH. The miR166-*EIN2* module may enhance ET signal pathway to interfere with the infection of *M. oryzae*. The siR109944-*FBL55* module may impede auxin signal pathway to promote *R. solani* infection. The miR160-*ARF18* and miR393-*TIR1*/*AFB* modules may act in auxin signal pathway. The miR156-*IPA1*/*SPL7* module may enhance GA signal pathway to promote the infection of *Xoo*. The miR159-*GAmyb* module may act in GA signal pathway. Arrows indicate positive regulation, circles indicate negative regulation

Rice (*Oryza sativa*) is the most important staple crop in Asian countries and feeds more than half of the world population. Rice production is essential for global food security. However, rice production is threatened by diverse diseases, such as rice blast caused by *Magnaporthe oryzae* (synonymous with *Pyricularia oryzae*), rice sheath blight caused by *Rhizoctonia solani* (*R. solani*), rice leaf blight caused by *Xanthomonas oryzae* pv. *oryzae* (*Xoo*), rice dwarf disease caused by *Rice dwarf virus* (RDV), rice stripe disease caused by *Rice stripe virus* (RSV). In return, rice employs an efficient defensive system to resist these pathogens. Both PTI and ETI are indispensable for rice immune system against fungal and bacterial pathogens (Wang et al. 2014), and antiviral RNA silencing is a crucial weapon for defense against viruses (Yang and Li 2018). Plants also exploit ETI against nematodes (Kandoth and Mitchum 2013). Moreover, hormone signals contribute to rice immunity against fungi, bacteria, viruses and insects (De Vleesschauwer et al. 2013). In recent years, an increasing number of reports demonstrate that sRNAs act as the critical regulators of gene expression to fine-tune rice immune responses to different pathogens and insect pests. Here, we summarize the recent progresses on the studies of rice sRNA pathway in the interactions of rice with pathogens and insect pests (Table 1 and Fig. 2).

**Table 1:** Characterized sRNAs involved in interactions of rice with pathogens and insect pests

sRNAs	Target genes	Biotic stressors	Literature
miR7695	*OsNramp6.8*	Resistance to *M. oryzae*	(Campo et al. 2013; Sanchez-Sanuy et al. 2019)
miR398b	*OsCSDs*	Resistance to *M. oryzae*	(Li et al. 2019b)
miR162a	*OsDCL1a*	Resistance to *M. oryzae*	(Li et al. 2020a)
miR166k-5p	*OsEIN2.1; OsEIN2.2*	Resistance to *M. oryzae* and *F. fujikuroi*	(Salvador-Guirao et al. 2018)
miR396	*OsGRFs*	Susceptible to *M. oryzae*, BPH; Resistance to *D. zeae*	(Chandran et al. 2018; Dai et al. 2019; Li et al. 2019a)
miR156	*OsSPLs*	Susceptible to *M. oryzae*, *Xoo*, BPH	(Ge et al. 2018; Liu et al. 2019; Zhang et al. 2020)
miR1873	*LOC_Os05g01790*;	Susceptible to *M. oryzae*	(Zhou et al. 2020)
miR167	*OsARFs*	Susceptible to *M. oryzae*	(Zhao et al. 2020)
miR319	*OsTCP21*; *OsGAmyb*	Susceptible to *M. oryzae*, RRSV	(Zhang et al. 2016a; Zhang et al. 2018b)
miR169	*OsNF-YAs*	Susceptible to *M. oryzae, Xoo*	(Li et al. 2017; Yu et al. 2018)
miR164a	*OsNAC11*; *OsNAC60*;	Susceptible to *M. oryzae*, *P. infestans*, *R. solani*	(Wang et al. 2018b)
miR444	*OsMADSs*	Susceptible to *M. oryzae*; Resistance to RSV	(Wang et al. 2016; Xiao et al. 2017)
miR171b	*OsSCL6-IIs*	Resistance to RSV	(Tong et al. 2017)
miR528	*L-ascorbate oxidase*;	Susceptible to RSV	(Wu et al. 2017)
TE-siR815	*OsWRKY45–1*	Susceptible to *Xoo*	(Zhang et al. 2016b)
siR109944	*OsFBL55*	Susceptible to *R. solani*	(Qiao et al. 2020)
miR168	*OsAGO1*	Susceptible to RSV	(Wu et al. 2015)
miR-14	*CsSpo*; *CsEcR*	Resistance to RSB	(He et al. 2019)

## Review

### Roles of Core Components of sRNA Pathway in Rice Immunity

Small RNA pathway possesses three types of core components, namely DCLs, AGOs, and RDRs. Rice genome encodes eight DCLs, nineteen AGOs and five RDRs. The DCLs include OsDCL1a, OsDCL1b, OsDCL1c, OsDCL2a, OsDCL2b, OsDCL3a, OsDCL3b/OsDCL5, and OsDCL4 (Kapoor et al. 2008; Margis et al. 2006). Different OsDCLs are responsible for the production of different sRNAs. For example, OsDCL1 and OsDCL3a are involved in the biosynthesis of 21- or 24-nt miRNAs (Liu et al. 2005; Yan et al. 2011). OsDCL3b/OsDCL5 and OsDCL4 are more involvement with the production of 21- or 24-nt siRNAs (Shi et al. 2007; Song et al. 2012a; Song et al. 2012b; Wu et al. 2010). OsDCL2 contributes to the accumulation of vsiRNAs derived from the *Oryza sativa* endornavirus (Urayama et al. 2010). Rice AGOs are phylogenetically classified into 6 clades, namely, AGO1, AGO4, AGO7, MEL1 clades, and two singleton clades. AGO1 clade includes OsAGO1a, OsAGO1b, OsAGO1c, OsAGO1d, and OsAGO10. AGO4 clade contains OsAGO4a, OsAGO4b, OsAGO15, and OsAGO16. AGO7 clade includes OsAGO2, OsAGO3, and OsAGO7. MEL1 clade includes MEL1, OsAGO11, OsAGO12, OsAGO13, and OsAGO14. The two singletons are OsAGO17 and OsAGO18 (Kapoor et al. 2008). OsAGO1a, OsAGO1b and OsAGO1c have a strong binding preference for miRNAs initiated with an uracil. In contrast, OsAGO4a and OsAGO16 predominantly bind to 24-nt miRNAs that are initiated with an adenine, whereas OsAGO4b mainly accommodates 24-nt miRNAs with an uracil or guanine (Wu et al. 2009; Wu et al. 2010). Rice RDRs include OsRDR1, OsRDR2, OsRDR3a, OsRDR3b, and OsRDR6 (Kapoor et al. 2008). OsRDR1, OsRDR2, and OsRDR6 are responsible for the accumulation of 21–24-nt siRNAs (Song et al. 2012b; Wu et al. 2010; Yang et al. 2008b). OsRDR1 also contributes to the production of several miRNAs, such as Osa-miR1859, Osa-miR395p, and Osa-miR820a (Wang et al. 2014)*.* To date, reports indicate that five *OsDCL*s, six *OsAGO*s, and two *OsRDR*s are involved in rice responses to different pathogens and insects.

Five *OsDCL*s have been reported to be involved in rice responses to *M. oryzae* and several viruses, including *OsDCL1*, *OsDCL2*, *OsDCL3a*, *OsDCL3b*/*OsDCL5*, and *OsDCL4*. *OsDCL1* positively regulates rice resistance to RSV, but compromises resistance to *M. oryzae*. The infection of *M. oryzae* promotes the expression of *OsDCL1* in the rice blast susceptible accession Lijiang xin Tuan Heigu (LTH), but reduces *OsDCL1* expression in the rice blast resistant accessions International Rice Blast Line Pyricularia-Kanto51-m-Tsuyuake (IRBLkm-Ts) and Yahui2115 (Li et al. 2020a). Knockdown of *OsDCL1* (*dcl1*) confers resistance to *M. oryzae* accompanying with high expression of multiple *PR* genes and two PTI related genes, *OsKS4* and *OsNAC4* (Li et al. 2020a; Zhang et al. 2015a). However, *dcl1* mutants are more susceptible to RSV associating with higher levels of RSV genomic RNAs and the coat protein transcripts (Yang and Li 2018). In contrast, activating the expression of *OsDCL1* results in suppressed expression of *OsPR1a* and *OsPBZ1*, and reduced accumulation of phytoalexin and tolerance to oxidative stress, thus compromising resistance against *M. oryzae* and *F. fujikuroi*, the causative agents of rice blast and bakanae disease, respectively (Salvador-Guirao et al. 2019). *OsDCL2* is down-regulated by the infection of *M. oryzae* and *Southern rice black-streaked dwarf virus* (SRBSDV), but significantly up-regulated by RSV infection (Du et al. 2011; Xu and Zhou 2017). Knockdown of *OsDCL2* (*dcl2*) abolishes the dsRNAs of the endogenous dsRNA virus *Oryza sativa* endornavirus. Besides, *dcl2* mutants exhibit compromised resistance to RSV (Urayama et al. 2010; Yang and Li 2018). Furthermore, *OsDCL3a*, *OsDCL3b*/*OsDCL5*, and *OsDCL4* are differentially responsive to *M. oryzae,* RSV, and RSBDV. Both *OsDCL3a* and *OsDCL3b*/*OsDCL5* are down-regulated by RSV infection, and *OsDCL4* is down-regulated by RBSDV infection, whereas both *OsDCL3a* and *OsDCL4* are up-regulated by *M. oryzae* infection (Du et al. 2011; Salvador-Guirao et al. 2019; Xu and Zhou 2017). Consistently, knockdown of either *OsDCL3a*, *OsDCL3b*/*OsDCL5* or *OsDCL4* leads to enhanced susceptibility to RSV (Yang and Li 2018). In conclusion, rice *DCL*s contribute to rice immunity against *M. oryzae* and viruses, and they play different roles in response to different parasitic organisms.

To date, six *AGO* genes are identified to be involved in rice responses to RSV or *M. oryzae*, including *OsAGO1a*, *OsAGO1b*, *OsAGO4a*, *OsAGO18*, *OsAGO2*, and *OsAGO11*. *OsAGO2* and *OsAGO11* are up-regulated in RSV- or RBSDV-infected rice plant, suggesting their potential roles in response to viruses (Du et al. 2011; Xu and Zhou 2017). *OsAGO18* cooperates with *OsAGO1* to enhance rice immunity against RSV. Inoculation with RSV improves the expression of *OsAGO1a*, *OsAGO1b*, and *OsAGO18* (Wu et al. 2015). Mutations in *OsAGO1a/b/c/d* (*ago1*) or *OsAGO18* (*ago18*) result in enhanced susceptibility to RSV, whereas overexpression of *OsAGO18* enhances resistance to RSV. Besides, OsAGO1a and OsAGO1b but not OsAGO18 protein could bind vsiRNAs to mediate antiviruses. Because OsAGO18 competes with OsAGO1 for binding miR168 during RSV infection, increase of OsAGO18 level leads to decreased the cleavage of *OsAGO1* transcripts mediated by miR168, thus keeping a high level of OsAGO1 protein and improving efficiency of antiviral RNA silencing to enhance rice immunity against RSV (Wu et al. 2015). *OsAGO4a* fine-tunes the expression of *PigmS* and *PigmR* from the *Pigm* locus to regulate rice blast resistance together with *OsRDR2* and *OsDCL3a* (Deng et al. 2017). Overexpression of *PigmR* confers a broad-spectrum resistance to *M. oryzae* with yield penalty, whereas overexpression of *PigmS* leads to susceptibility to *M. oryzae* but increased yield under blast disease-free conditions. PigmS interacts with PigmR to attenuate immune responses mediated by PigmR via abrogating the formation of PigmR homodimer. *OsAGO4a* precisely regulates the expression of *PigmS* via mediating DNA methylation of two tandem miniature transposons (MITE1, MITE2) located in the promoter of *PigmS*. As a result, high levels of methylation in MITE1 and MITE2 lead to repressed expression of *PigmS* and releases of *PigmR*, thus resulting in a strong immunity at vegetative stage; whereas reduced methylation levels in MITE1 and MITE2 in pollen lead to enhanced expression of *PigmS*, thus rescuing seed production to counteract yield penalty induced by *PigmR* (Deng et al. 2017). Therefore, *OsAGO1a*, *OsAGO1b* and *OsAGO18* positively regulate rice immunity against viruses, and *OsAGO4* plays a critical role in the trade-off between immunity against *M. oryzae* and yield productivity.

RDRs are mainly involved in antiviral functions. *OsRDR1* and *OsRDR6* have been reported to regulate resistance against RSV and RDV. The expression of *OsRDR1* is enhanced following the inoculation with SRBSDV and RDV (Du et al. 2011; Xu and Zhou 2017). *OsRDR1* is required for antiviral RNA silencing triggered by the ssRNA virus *Brome mosaic bromovirus*, but not the ssDNA virus *wheat dwarf geminivirus* (Chen et al. 2010). Moreover, knockout of *OsRDR1* reduces rice resistance against RSV accompanying with increased accumulation of RSV RNA and CP proteins (Wang et al. 2016). Similarly, silencing of *OsRDR6* (*rdr6*) is more susceptible to RSV and RDV. Moreover, *rdr6* mutants show a reduced vsiRNAs derived from RSV and RDV, implying the important role of *RDR6* in producing vsiRNAs (Hong et al. 2015b; Jiang et al. 2012). Whereas, overexpression of *OsRDR6* (OXRDR6) shows a comparable resistance with wild type (WT) plants. Further studies show that RDV-infection reduces the transcripts of *OsRDR6* and abolishes the protein accumulation of RDR6 in the *OXRDR6* transgenic plants (Hong et al. 2015b), suggesting that *OsRDR6* may be targeted by viruses to facilitate infection. Indeed, some viral proteins have been proposed to disturb with the functions of *OsRDR6*. RDV Pns10 protein could repress the expression of *OsRDR6*, and may bind vsiRNAs to prevent the initiation of antiviral RNA silencing (Ren et al. 2010). Additionally, the P6 protein encoded by *Rice yellow stunt rhabdovirus* could interact with OsRDR6 thus interfering with its function in the production of vsiRNAs, eventually leading to suppressed rice immunity (Guo et al. 2013).

In conclusion, some core components of sRNA pathway are essential for antiviral RNA silencing signals, such as *OsDCL1*, *OsDCL2*, *OsDCL3a*, *OsDCL3b*/*OsDCL5*, *OsDCL4*, *OsAGO1*, *OsAGO18*, *OsRDR1*, and *OsRDR6*. Besides, *OsDCL1* negatively, but *OsAGO4a* positively regulates rice resistance against *M. oryzae*. *OsRDR6* is targeted by several viruses to disturb with the antiviral RNA silencing. As the core components of sRNA pathway, *DCL*s, *AGO*s and *RDR*s may work synergistically or antagonistically to fine-tune rice responses to different pathogens. Therefore, it is worthy to further study the roles of the biotic-responsive members of *OsDCL*s, *OsAGO*s, and *OsRDR*s in rice immunity.

### Small RNAs in Rice-*M. oryzae* Pathological System

Rice-*M. oryzae* pathological system has become an ideal model for the study of plant-fungus interactions. More than 70 miRNAs respond to *M. oryzae* or its elicitors (Li et al. 2019c). Among these miRNAs, five have been identified as positive regulators in rice blast resistance, namely miR160a, miR162a, miR166k-h, miR398b, and miR7695, whereas eight have been identified as negative regulators, namely miR156, miR164a, miR167d, miR169a, miR319b, miR396, miR1873, and miR444b.2(Chandran et al. 2018; Li et al. 2020a; Li et al. 2019b; Li et al. 2014; Li et al. 2017; Salvador-Guirao et al. 2018; Sanchez-Sanuy et al. 2019; Wang et al. 2018b; Xiao et al. 2017; Zhang et al. 2018b; Zhang et al. 2020; Zhao et al. 2020; Zhou et al. 2020). Obviously, many miRNAs play a role in regulating rice-*M. oryzae* interactions.

Four miRNAs are well-studied in regulating rice-*M. oryzae* interactions. First, miR7695 is a rice-specific miRNA that positively regulates rice blast resistance via suppressing the expression of *OsNramp6.8*, an iron transporter that negatively regulates rice immunity (Campo et al. 2013; Perisperis et al. 2017). Overexpression of miR7695 results in increased iron accumulation, enhanced innate immune responses, and increased accumulation of phytoalexins. Further studies revealed that high concentration of iron improves the expression of *OsCPS2* and *OsCPS4*, which function in the first cyclization steps of phytoalexin biosynthesis, suggesting that miR7695 may promote phytoalexins accumulation via controlling the transport of iron (Campo et al. 2013; Sanchez-Sanuy et al. 2019; Toyomasu et al. 2015). Second, miR398b is a well-elucidated positive regulator in rice resistance against *M. oryzae*. miR398b targets several members of the superoxide dismutase (SOD) family genes, including *copper-binding SOD1* (*OsCSD1*), *OsCSD2, OsSODX*, and *chaperone of CSD* (*OsCCSD*). SODs catalyze the reduction of superoxide radicals (O·2^−^) into hydrogen peroxide (H_2_O_2_), which is an indispensable component in plant immunity (Kaur et al. 2014; Mittler et al. 2011). The transgenic lines overexpressing miR398b, and mutants of *OsCSD1*, *OsCSD2*, and *OsSODX* display enhanced resistance against *M. oryzae* accompanying with enhanced SOD enzyme activity and increased H_2_O_2_ accumulation. Conversely, blocking miR398, or overexpression of *OsCSD2*, or mutation of *OsCCSD* facilitates the infection of *M. oryzae*, which is associated with suppressed SOD enzyme activity and decreased H_2_O_2_ accumulation (Li et al. 2019b). Therefore, miR398b coordinately controls the SODs enzyme activity via manipulating different target genes to enhance the production of H_2_O_2_ (Li et al. 2019b). Different from miR7695 and miR398b, both miR164a and miR319b facilitate *M. oryzae* infection. miR164a targets two transcription factor genes, *OsNAC11* and *OsNAC60*, which belongs to NAC (NAM/ATAF/CUC) transcription factor family that play critical roles in plant development and stress-induced responses (Puranik et al. 2012). Overexpressing miR164a or silencing *OsNAC60* leads to repressed expression of *OsNPR1* and *OsPBZ1*, two defense marker genes for innate immunity and SA signaling pathway (Lu et al. 2015). In contrast, overexpression of *OsNAC60* in *Nicotiana benthamiana* triggers strong autoimmunity phenotypes that could be abolished by co-expressing of miR164a. Furthermore, overexpression of miR164a leads to compromised host resistance to *Phytophthora infestans* and *R. solani*, two destructive filamentous pathogens in potato, tomato and rice, respectively, indicating that miR164a may play a conserved role in plant immunity against different filamentous pathogens (Wang et al. 2018b). Similarly, miR319 also negatively regulates rice resistance to *M. oryzae*. Overexpression of miR319b (*OX319b*) or interfering with the expression of *OsTCP21* (*TCP21i*), one target gene of miR319, promotes infection of *M. oryzae*, which is associated with suppressed induction of basal immune responses and impaired JA signal. The plant hormone JA is necessary for resistance against hemi-biotrophic and necrotrophic pathogens (De Vleesschauwer et al. 2013). In contrast, overexpression of Os*TCP21* impedes infection of *M. oryzae* presumably due to enhanced basal immune responses and JA signal. Considering miR319b is up-regulated during infection, miR319b may be manipulated by *M. oryzae* to suppress rice immunity through repressing basal immune responses and JA signal (Zhang et al. 2018b).

Besides the miRNA regulatory modules mentioned above, quite a few miRNA regulatory modules have been functionally characterized in rice immunity against *M. oryzae* (Table 1). The negative regulatory modules generally impair the accumulation of H_2_O_2_ and the induction of defense-related genes in transgenic lines overexpressing miRNAs or mutants of target genes, such as miR167d-*OsARF12*, miR396a/c/d/h-*OsGRF6/7/8/9*, miR169a-*OsNF-YAs*, miR444b.2-*OsMADS*, miR1873-*Os05g01790*, and miR156-*OsSPL14* (Chandran et al. 2018; Li et al. 2017; Xiao et al. 2017; Zhang et al. 2020; Zhao et al. 2020; Zhou et al. 2020). Conversely, the positive regulatory modules enhance defense responses, such as miR162a-*OsDCL1* and miR166k-*OsEIN2.1* modules (Li et al. 2020a; Salvador-Guirao et al. 2018). Furthermore, MITE1- and MITE2-derived siRNAs trigger DNA methylation of the promoter of *PigmS* and repress its expression, thus to positively regulate rice blast resistance (Deng et al. 2017) (Table 1 and Fig. 2).

In addition to sRNA pathway in rice, sRNA pathway of *M. oryzae* also contributes to rice-*M. oryzae* interactions. Several studies have identified some core components of sRNA pathway and a lot of miRNA-like RNAs (milRNAs) in *M. oryzae*. *MoDCL2*, *MoRdRP2*, and *MoAGO3* are core components of sRNA pathway in *M. oryzae*. Mutation of *MoDCL2*, *MoRdRP2*, and *MoAGO3* results in defects in fungal growth, development, and virulence, suggesting their indispensability in the pathogenicity of *M. oryzae* (Raman et al. 2017; Raman et al. 2013). Besides, there are 171 non-redundant milRNAs identified to be differentially expressed among wild type strain, and the appressorium-defective strains Δ*Morgs1*, Δ*Morgs3*, and Δ*Morgs7* (Li et al. 2020b), implying their roles in appressorial formation and virulence. Indeed, overexpression of milR236 leads to delayed appressorium formation and impaired virulence. milR236 is one of the differentially expressed milRNAs, its overexpression leads to suppression of the target gene *MoHat1*, a histone acetyltransferase that is required for the formation of appressorium (Li et al. 2020b). Furthermore, there are 366 sRNAs up-regulated in *M. oryzae* during infection and fourteen of them are predicted to target rice genes (Zhang et al. 2019). This report implies the existence of cross-kingdom RNA silencing in rice-*M. oryzae* interactions.

The cross-kingdom RNA silencing has been discovered in the interaction between *Arabidopsis* plants and *Botrytis cinerea* (*B. cinerea*), and in the interaction between cotton and *Verticillium dabliae*. For example, Bc-siR3.2 is up-regulated in *B. cinerea*-infected plants and targets two host genes, *AtMPK2* and *AtMPK1*. Ectopically expressing of Bc-siR3.2 in *Arabidopsis* leads to more susceptible to *B. cinerea* and significantly reduces the mRNA levels of *AtMPK2* and *AtMPK1*, whose double mutants show enhanced susceptibility to *B. cinerea* (Wang et al. 2017b; Weiberg et al. 2013). In return, plants also export sRNAs into pathogens to interfere with *B. cinerea* infection. During infection of *B. cinerea*, *Arabidopsis* cells secrete exosome-like extracellular vesicles to deliver sRNAs into fungal cells of *B. cinerea*. For example, the two tasiRNAs TAS1c-siR483 and TAS2-siR453, target three genes *Bc-Vsp51*, *Bc-DCTN1*, and *Bc-SAC1*, which are involved in vesicle-trafficking pathways important for fungal virulence (Cai et al. 2018). Therefore, cross-kingdom RNA silencing in rice-*M. oryzae* interactions could be a future research focus.

### Small RNAs in Rice-Viruses Pathological System

Rice production are challenged by many different viruses, such as *Rice stripe virus* (RSV), *Rice dwarf virus* (RDV), *Rice ragged stunt virus* (RRSV), *Rice black-streaked dwarf virus* (RBSDV), *Southern rice black-streaked dwarf virus* (SRBSDV), *Rice tungro bacilliform virus* (RTBV), and *rice tungro spherical virus* (RTSV). A lot of sRNAs has been identified during different viruses infection, including RSV, RDV, RBSDV, SRBSDV, RTBV, and RTSV (Du et al. 2011; Guo et al. 2015; Guo et al. 2012; Lian et al. 2016; Xu and Zhou 2017; Yang et al. 2016; Zarreen et al. 2018). Specifically, the roles of rice miRNAs are the most studied in the rice-RSV interaction. Two miRNAs act as positive regulators and one miRNA acts as a negative regulator in the regulation of anti-RSV responses. RSV is a brown planthopper (BPH)-transmitted single-stranded RNA virus (Wang et al. 2008). A series of rice miRNAs are differentially expressed at the RSV-infected plants (Du et al. 2011; Guo et al. 2012; Lian et al. 2016; Yang et al. 2016). Among these miRNAs, miR444 and miR171 are reported to positively regulate rice resistance to RSV, whereas miR528 negatively regulates resistance to RSV. miR444 is induced in RSV-infected plants, leading to suppressed expression of three target genes, namely *OsMADS23, OsMADS27a*, and *OsMADS57* that belong to the MADS transcription factor family. Plant MADS box proteins regulate genes expression by binding the CArG motif, one conserved motif that exists in the promoter of *OsRDR1* (de Folter and Angenent 2006). Further studies pointed that OsMADS23, OsMADS27a, and OsMADS57 interact with each other to form homodimers or heterodimers to repress the expression of *OsRDR1*, whose knockout mutants exhibit enhanced susceptibility to RSV. Therefore, miR444-*OsMADS* modules enhance antiviral ability via promoting antiviral RNA silencing signal (Wang et al. 2016). Conversely, RSV-infection represses the accumulation of miR171b. Blocking the function of miR171b leads to phenotypes similar to those of RSV-infected rice plants, containing a stunted growth and reduced chlorophyll content in leaves. Moreover, overexpression of miR171b enhances resistance to RSV accompanying with repressed expression of three target genes, including *OsSCL6-IIa*, *OsSCL6-IIb*, and *OsSCL6-IIc* (Tong et al. 2017). Different from miR444 and miR171b, miR528 negatively regulates rice resistance to RSV. During RSV infection, miR528 is sequestered by OsAGO18, thus elevating the expression of *L-ascorbate oxidase* (*AO*), one target gene of miR528 (Wu et al. 2015; Yao et al. 2019). AO protein regulates the apoplast redox state in plants by oxidizing L-ascorbic acid (AsA), leading to decelerate the detoxification of reactive oxygen species (ROS) (Mittler et al. 2011; Pignocchi et al. 2006). Mutation of miR528, or overexpression of *AO* improves basal ROS levels as a result of enhanced rice immunity. Conversely, overexpression of miR528 impedes antiviral ability presumably due to reduced basal ROS levels (Wu et al. 2017).

In addition to participate in the rice-RSV interaction, miRNAs are also found to respond to other viruses. There are 14 miRNAs in leaves and 16 miRNAs in roots differentially respond to RBSDV-infection. Among them, five miRNAs show a similar expression pattern, including miR166a-f, miR169h-m, miR408, miR827, and miR1428e; and the other miRNAs display a diverse expression patterns, including miR394, miR397, miR530, miR162, miR398, miR1862d, miR159ab, miR171, and miR1432 (Sun et al. 2015). Moreover, a total of 54 responsive rice miRNAs are identified after inoculating with RTBV and RTSV. Among them, miR5493, miR159e, and miR1875 are up-regulated, whereas down-regulated miRNAs are belonging to the families of miR167, miR164, miR156, miR815, miR171, miR444, miR166, miR1439, miR396, miR169, miR818, miR172, and miR408. In contrast, RDV-infection barely disturbs the homeostasis of rice miRNAs, only a few miRNAs are respond to RDV, including miR167a, miR171, miR1863, and miR393 (Du et al. 2011). These reports suggest that rice miRNAs are more involved in regulating rice responses to RSBDV, RTBV, and RTSV than to RDV. Furthermore, miR319 has also been identified to negatively regulate anti-RRSV resistance. RRSV is a double-stranded RNA virus that is transmitted by BPH (Ling et al. 1978). miR319 targets four *TCP* genes, including *OsPCF5*, *OsPCF6*, *OsPCF8* and *OsTCP21*. miR319 is induced during RRSV infection associating with a significant reduction of *OsTCP21* expression. Overexpressing miR319 (*OX319*) or silencing *OsTCP21* (*TCP21* IR) results in plant phenotypes similar to RRSV-infected rice plants that showed stunted growth, excess tillering, and dark green and crinkle leaves. Besides, *OX319* and *TCP21* IR transgenic plants support more RRSV duplication accompanying with decreased JA content. Conversely, overexpression of *OsTCP21* results in decreased RRSV proliferation associating with increased JA content (Martintrillo and Cubas 2010; Zhang et al. 2016a).

Furthermore, viruses are spread by the viral vector insects, some of the viral vector insects also cause significant loss on rice production independent of viruses. The brown planthopper (BPH) is a rice-specific herbivore and causes direct mechanical damage to rice phloem using the stylet (Sōgawa 1982). A total of 55 differentially expressed miRNAs (DEMs) are identified between the BPH resistant rice BPH6G and wild type following the invasion of BPH. Among these DEMs, 29 are oppositely expressed in BPH6G and wild type, containing miRNAs from families of miR169, miR166, miR160, miR156, miR396, miR319, and miR1861, indicating that these DEMs may contribute to the immune responses triggered in BPH6G (Tan et al. 2020). Moreover, miR156 and miR396 are further proved to negatively regulate rice resistance against BPH. Silencing of miR156 improves tolerance to BPH, and leads to a suppressed expression of multiple JA biosynthesis genes and decreased accumulation of JA/JA-Ile. Because the JA signal pathway is considered to negatively regulate BPH resistance in rice, miR156 acts as a negative regulator in rice resistance against BPH via promoting JA pathway (Dai et al. 2019; Li et al. 2015; Zhou et al. 2009). miR396 is a well-studied negative regulator in antiviruses and targets 12 growth regulating factor (GRF) genes (Gao et al. 2015). Overexpression a target mimic of miR396 (MIM396) leads to enhanced resistance to BPH accompanying with down-regulated JA signal genes *OsCoia* and *OsCoib* and up-regulated SA marker gene *OsNPR1* (Dai et al. 2019). Consistently, transgenic plants overexpressing *OsGFR8*, one target gene of miR396, results in phenotypes similar to those of MIM396. Later studies found that *OsGRF8* directly activates the expression of *OsF3H*, one gene that responses to BPH and is involved in flavonoid biosynthesis (Wang et al. 2012). Flavonoid is an important secondary metabolite against biotic or abiotic stress (Pourcel et al. 2007). Overexpression of *OsF3H* enhances flavonoid contents and improves tolerance to BPH; whereas silencing of *OsF3H* compromises resistance to BPH, indicating that miR396-*OsGRF8* module may be utilized by BPH to suppress rice immunity via manipulating downstream hormone signal and flavonoid biosynthesis (Dai et al. 2019). Intriguingly, JA signal exhibits two-faced roles between rice-viruses interaction and rice-BPH interaction. Usually, JA is considered to be a positive regulator of virus resistance whereas a negative regulator of BPH resistance in rice. Given that some viruses are transmitted with the help of BPH in field, many questions can be research foci in the future. For example, how do plants/viruses avoid or exploit multi-faceted roles of JA during infection? Whether do sRNAs play an important role in fine-tuning the contradictory relationship during infection?

Moreover, the sRNAs of viruses and insects also contribute to the interactions of rice with viruses and insects. After inoculating with SRBDV, 366 vsiRNAs are detected in the 14-dpi samples and 28-dpi samples. The vsiRNAs are mostly in 21- and 22-nt, implying that OsDCL2 and OsDCL4 are the predominant enzymes for the biogenesis of SRBDV-derived siRNAs. A total of 151 vsiRNAs are predicted to target 844 rice genes related to disease/stress responses and RNA silencing components, suggesting the contribution of vsiRNAs in viral pathogenicity (Lan et al. 2018; Xu and Zhou 2017). Besides, miR-14 is a conserved miRNA in rice stem borers (RSB) and planthoppers. miR-14 of *Chilo suppressalis* (*Csu-miR-14*) targets *CsSpo* and *CsEcR*, two genes that act in ecdysone signal network. Overexpression of *Csu-miR-14* in RSB leads to abnormal development. Consistently, overexpression of *Csu-miR-14* (*OX-14*) in rice leads to enhanced resistance to RSB (He et al. 2019). Feeding RSB with *OX-14* transgenic plants causes developmental defects and high mortality. Therefore, miR-14 could be a potent candidate tool in engineering resistance against RSB in rice.

### The Involvement of sRNAs in Other Rice Diseases

In addition to rice blast disease and rice virus diseases, sRNAs are also involved in regulating immune responses to the other rice diseases, such as rice bacterial blight, rice foot rot, and rice sheath blight disease.

Rice leaf blight is one of the most devastating bacterial diseases of rice caused by *Xanthomonas oryzae* pv. *oryzae* (*Xoo*). Several sRNAs are identified to be differentially expressed between the *Xoo*-susceptible accession MDJ8 and the *Xoo*-resistant accession Rb49 that carries the *Xoo*-resistance genes *Xa3*/*Xa6* (Hong et al. 2015a), suggesting that these sRNAs may contribute to the *Xa3*/*Xa6*-mediated resistance. Among these *Xoo*-responsive sRNAs, miR156, miR169o, and TE-siR815 are identified as negative regulators in rice resistance against *Xoo* (Liu et al. 2019; Yu et al. 2018; Zhang et al. 2015b). miR156 is induced following the invasion of *Xoo*, and overexpression of miR156 boosts *Xoo* infection accompanying with decreased expression of *OsPR1b*, *OsPR1a*, and *OsWRKY45*. Conversely, overexpression of a miR156 target mimic (MIM156), or *OsIPA1*, or *OsSPL7*, the target genes of miR156, enhances rice resistance to *Xoo* associating with improved expression of *OsPR1b*, *OsPR1a*, and *OsWRKY45* (Liu et al. 2019). Furthermore, OsIPA1 and OsSPL7 block GA signaling pathway via stabilizing OsSLR1, a repressor of GA signaling pathway. Because GA promotes rice susceptibility to *Xoo*, and mutation of *OsSLR1* in MIM156 background reduces resistance to *Xoo*, miR156 most likely acts as a negative regulator in rice immunity against *Xoo* via promoting GA signal pathway and suppressing the basal immune responses (Liu et al. 2019; Yang et al. 2008a). In contrast, miR169o is suppressed following the inoculation of *Xoo* (Yu et al. 2018). Overexpression of miR169o causes a much greater lesion associating with suppressed expression of *OsPR1b*, *OsPR10a*, and *OsPAL*. Consistently, overexpression of the target genes of miR169o leads to enhanced expression of *OsPR1b*, *OsPR10a* and *OsPAL*, implying that miR169o may suppress the innate immune responses to negatively regulate rice resistance against *Xoo* (Yu et al. 2018). Similar to miR156 and miR169o, TE-siR815 also negatively regulates rice immunity against *Xoo*. TE-siR815 is derived from the intron region of *WRKY45–1* locus. *WRKY45* has two allelic loci, including *WRKY45–1* and *WRKY45–2.* Overexpression of *WRKY45–2* enhances rice resistance to *Xoo*, while overexpression of *WRKY45–1* leads to susceptibility to *Xoo*. Further studies found that TE-siR815 targets and represses the expression of *OsST1,* an important downstream component in *WRKY45*-mediated resistance against *Xoo* (Zhang et al. 2016b). Obviously, TE-siR815 blocks the *OsWRKY45*-mediated immune responses to facilitate the proliferation of *Xoo*.

Rice sheath blight also ranks one of the most devastating diseases of rice caused by the fungal pathogen *Rhizoctonia solani*. Many miRNAs are differentially expressed between the rice sheath resistant line YSBR1 and the susceptible line Xudao3 following the inoculation of *R. solani*, including osa-miR398a, osa-miR1881, osa-miR530, osa-miR444, osa-miR812, osa-miR1861, osa-miR3980, osa-miR531f families, osa-miR171f-5p, osa-miR2863a, osa-miR3979-3p, osa-miR1428e-3p, and osa-miR156 (Cao et al. 2020). Moreover, 14 long siRNAs (lsiRNAs) in length from 25- to 30-nt and several sRNAs are also identified to be responsive to *R. sonali*. Among them, some siRNAs-target modules show a tight conversely co-expression relationship, including lsiRNA51031*-Os08g15322*, lsiRNA73750*-Os09g14490*, lsiRNA118183*-Os08g06220*, lsiRNA194568*-Os06g38990*, miR167h*-OsARF8,* miR171a*-Os02g44360*, and miR160c*-OsARF10*, suggesting their involvement in regulating rice response to *R. solani* (Niu et al. 2018; Qiao et al. 2020). Among these differentially expressed sRNAs, siR109944 is further proved as a negative regulator in rice resistance against *R. solani*. siR109944 is derived from a precursor containing DNA transposons and targets *OsFBL55* that encodes a protein containing a typical TIR domain and sharing 65.57% similarity with the IAA receptor OsTIR1 (Dharmasiri et al. 2005; Yu et al. 2013). siR109944 is repressed upon the infection of *R. solani*. Overexpression of siR109944 facilitates the growth of *R. solani* companying with reduced IAA accumulation, whereas overexpression of *OsFBL55* significantly suppresses *R. solani* growth associating with increased IAA accumulation. Consistently, IAA application improves resistance to *R. solani*, suggesting that IAA positively regulates rice sheath blight resistance and siR109944 manipulates auxin signal pathway to negatively regulate rice resistance to *R. solani* (Qiao et al. 2020). Furthermore, sRNAs derived from *R. solani* are also found to be involved in rice-*R. solani* interaction. A total of 109 conserved milRNAs and 68 novel milRNAs are identified during *R. solani* infection in the AG1 IA strain and they are predicted to target genes involved in pathogenicity (Lin et al. 2016).

Rice foot rot is another emerging bacterial disease caused by *Dickeya zeae* (*D. zeae*). A total of 79 miRNAs is differentially expressed following the infection of *D. zeae* in a foot rot resistant accession Nanjing 40, including miR2118, miR393, miR166, miR171, miR156, miR159, miR396. Among them exists 29 co-expressed miRNA-target modules including miR396-*OsGRFs*, indicating their involvement in rice-*D. Zeae* interaction. Furthermore, overexpression of miR396f enhances rice resistance to *D. zeae* associating with a milder symptom in roots (Li et al. 2019a).

### Small RNA Interconnects Hormone Signaling and the Other Non-coding RNAs

Small RNA seems to post its roles largely via a network of transcription factors that may act in different hormone signaling pathways. On one hand, rice sRNAs could manipulate hormone signal pathways to confer or compromise rice immunity against *M. oryzae*, *Xoo*, *R. solani*, or viruses (Fig. 2). For examples, miR319-*OsTCP21* module negatively regulates rice resistance against *M. oryzae* and RRSV via blocking JA signal pathway; miR156-*OsSPL7*/*OsIPA1* module negatively regulates rice resistance via promoting GA signal pathway (Liu et al. 2019; Zhang et al. 2016a; Zhang et al. 2018b). Whereas, miR166k-*OsEIN2* module positively regulates resistance to *M. oryzae* by activating ET signal pathway (Salvador-Guirao et al. 2018). On the other hand, hormone signal pathways could regulate the sRNA pathway in return. For example, the expression of pre-miR166k-166 h is rapidly increased following the treatment with ethylene precursor ACC (1-aminocyclopropane-1-carboxylic acid), which suggests a positive regulation loop between miR166k and ethylene signal pathway. Besides, several *M. oryzae*-responsive miRNAs carry the hormone-related cis-elements in the promoters. For example, the classical Auxin responsive element TGTCTC exists in the promoters of miR167d and miR160a, which both target the ARF family members that are downstream components of Auxin signal pathway. Furthermore, recent reports pointed that JA positively regulates the expression of *OsAGO18* and enhances rice antiviral activity (Wu et al. 2015; Yang et al. 2020b). Therefore, the sRNA pathway may manipulate the hormone signal to regulate rice resistance against the pathogenic organisms, and at the same time, the expression of some sRNAs may be controlled by the transcription factors that act downstream of some specific hormone signal.

In addition to sRNAs, other non-coding RNAs, such as long non-coding RNAs (lncRNAs) and circular RNAs (circRNAs), have been emerging to take part in regulating rice-pathogen interactions (Wang et al. 2017a). LncRNAs are longer than 200-nt and could execute their function to interact with sRNAs in two ways: (1) act as the target mimic of miRNAs to block miRNAs from binding their target genes, and (2) serve as sRNAs precursors to produce sRNAs (Wang et al. 2017a). For examples, *AtIPS1* acts as a target mimic of miR399 to protect miR399’s target gene *AtPHO2* from the cleavage by miR399 under phosphate deficiency condition (Franco-Zorrilla et al. 2007). In *Blumeria graminis f. sp. tritici*-infected wheat, TalncRNA5 and TapmlnRNA19 are identified as the precursors of miR2004, and TapmlnRNA8 is the precursor of miR2066 (Xin et al. 2011). *Xoo* infection triggers differential expression of 576 lncRNAs and these lncRNAs are predicted to target genes enriched in JA signal pathway. One lncRNA, ALEX1, is proved to up-regulate the expression of JA synthesis genes and JA signal genes, thus positively regulating resistance to *Xoo* (Yu et al. 2020). Except for responding to *Xoo*, lncRNAs are also responsive for *D. zeae*. A total of 4709 lncRNAs are identified upon *D. zeae* infection, and their predicted target genes are involved in multiple signal pathways related to defense responses (Li et al. 2018a). CircRNAs are formed by pre-mRNAs through back-splicing in which the upstream 3′ splicing acceptor site is joined to the downstream 5′ splicing donor site (Ashwal-Fluss et al. 2014). Similar to lncRNAs, circRNAs could block the function of miRNAs by acting as the miRNA sponges (Li et al. 2018b). In rice, the amount of circRNAs is more abundant in the rice blast resistance accession IRBLkm-Ts than the rice blast susceptible accession LTH, and a total of 636 circRNAs specifically expressed after the infection of *M. oryzae*, indicating the involvement of circRNAs in rice responses to *M. oryzae* (Fan et al. 2020). Moreover, circR5g05610 is further proved to enhance rice immunity against *M. oryzae* (Fan et al. 2020). Given that lncRNAs and circRNAs are capable of disturbing miRNAs function by competing miRNA with miRNA targets, or producing miRNAs, or acting as miRNA sponges via masking miRNA binding sites (Huang et al. 2017; Li et al. 2018b), it is possible that sRNAs, lncRNAs, and circRNAs could cooperate or antagonize with each other to mediate rice immunity.

## Conclusions

The core components of sRNA pathway and varieties of sRNAs act as critical regulators of gene expression to fine-tune rice immunity against parasitic organisms, including fungi, bacteria, viruses, and insects. Some specific sRNA-target modules are involved and may act consistently or conversely in the interactions of rice with different biotic stressors. For examples, miR396a/c/d/h-*OsGRF6*/*OsGRF7*/*OsGRF8*/*OsGRF9* modules negatively regulate resistance to *M. oryzae* but miR396-*OsGRF8* module acts as a negative regulator in rice resistance against BPH. The miR319-*OsTCP21* module negatively regulates rice resistance to RRSV and *M. oryzae*. miR444-*OsMADS23*/*OsMADS27a*/*OsMADS57* modules positively regulate rice resistance to RSV, but overexpression of miR444 leads to more susceptible to *M. oryzae*. Besides, some sRNAs also coordinate responses to biotic stressors with the regulation of growth and development. For examples, miR156-*OsIPA1* module negatively regulates rice resistance to *M. oryzae* and it also regulates seed dormancy and germination; miR396-*OsGRFs* modules negatively regulate rice resistance to *M. oryzae* and they also regulate plant height, leaf morphology and seed size (Guo et al. 2016; Tang et al. 2018; Zhang et al. 2018a; Zhang et al. 2020). Therefore, sRNA pathway may act as a part of innate immunity to tightly coordinate rice growth, development, and immunity, thus could be used as a candidate to balance the trade-off between yield and resistance in rice breeding.

Currently, our understanding is fragmentary on the roles of sRNA pathway in the interactions of rice with pathogens and insect pests and limited to several important pathogens, such as *M. oryzae* and some viruses. Even to these pathogens, many more pathogen-responsive sRNAs need further functional characterization. Because some sRNAs that function in rice response to one pathogen may also function in response to the other, priority of future research should be focused on those sRNAs characterized in one rice-pathogen interaction. For example, miR168, miR171, and miR528 act in rice-virus interaction, they might also function in rice-*M. oryzae* or rice-*Xoo* interaction. Consistently, miR7695 and siR109944 contribute to rice-fungi interactions, they might function in rice-virus interaction. Priority may also be focused on identifying the upstream regulators that determine the accumulation of sRNAs during the invasion of biotic stressors and on dissecting the down-stream components that relay the regulatory signal mediated by the sRNA-target modules. Besides, no studies have identified sRNAs that function in cross-kingdom RNA silencing during infection in rice. Thus, it is worthy to explore whether sRNAs derived from rice or biotic stressors contribute to cross-kingdom regulation.

## Data Availability

Not applicable.

## Abbreviations

PTI: pattern-triggered immunity
HR: hypersensitive response
DCL: Dicer-like
RDR: RNA-dependent RNA polymerase
AGO: ARGONAUTE
PTGS: post-transcriptional gene silencing
TGS: transcriptional gene silencing
vsiRNA: virus-derived siRNA
VSRs: suppressors of RNA silencing
siRNAs: small interfering RNAs
pri-miRNA: primary miRNA
miRNA: microRNA
PoI II: RNA polymerase II
pre-miRNA: precursor miRNA
HYL1: HYPONASTICLEVES1
SE: SERRATE
lncRNA: long non-coding RNA
*M. oryzae*: *Magnaporthe oryzae*
*P. infestans*: *phytophthora infestants*
*R. solani*: *Rhizoctonia solani*
H_2_O_2_: hydrogen peroxide
Rbohs: respiratory burst oxidase homologs
SOD: superoxide dismutase
ARF: auxin responsive factor
GRF: growth regulating factor
NF-YA: nuclear transcription factor Y
SPL: SQUAMOSA promoter-binding protein-like transcription factor
EIN2: ethylene-insensitive 2
FBL55: F-Box domain and LRR-containing protein 55
RSV: *Rice stripe virus*
RDV: *Rice dwarf virus*
RBSDV: *Rice black streaked dwarf virus*
SRBSDV: *Southern rice black-streaked dwarf virus*
Nramp6: natural resistance-associated macrophage protein 6
ssRNA: single-stranded RNA
dsRNA: double-stranded RNA
AO: L-ascorbate oxidase
BPH: brown planthopper
Xoo: *Xanthomonas oryzae* pv. *oryzae*
*D. zeae*: *Dickeya zeae*
JA: jasmonic acid
SA: salicylic acid
GA: gibberellin acid
ET: ethylene
